# A systematic review of intimate partner violence interventions focused on improving social support and/ mental health outcomes of survivors

**DOI:** 10.1371/journal.pone.0235177

**Published:** 2020-06-25

**Authors:** Emilomo Ogbe, Stacy Harmon, Rafael Van den Bergh, Olivier Degomme

**Affiliations:** 1 International Centre for Reproductive Health, Ghent University, Ghent, Belgium; 2 Georgia State University Alumna, Atlanta, Georgia, United States of America; 3 Médecins Sans Frontières-Operational Centre Brussels, Brussels, Belgium; Ben-Gurion University of the Negev Faculty of Health Sciences, ISRAEL

## Abstract

**Background:**

Intimate partner violence (IPV) is a key public health issue, with a myriad of physical, sexual and emotional consequences for the survivors of violence. Social support has been found to be an important factor in mitigating and moderating the consequences of IPV and improving health outcomes. This study’s objective was to identify and assess network oriented and support mediated IPV interventions, focused on improving mental health outcomes among IPV survivors.

**Methods:**

A systematic scoping review of the literature was done adhering to PRISMA guidelines. The search covered a period of 1980 to 2017 with no language restrictions across the following databases, Medline, Embase, Web of Science, PROQUEST, and Cochrane. Studies were included if they were primary studies of IPV interventions targeted at survivors focused on improving access to social support, mental health outcomes and access to resources for survivors.

**Results:**

337 articles were subjected to full text screening, of which 27 articles met screening criteria. The review included both quantitative and qualitative articles. As the focus of the review was on social support, we identified interventions that were i) focused on individual IPV survivors and improving their access to resources and coping strategies, and ii) interventions focused on both individual IPV survivors as well as their communities and networks. We categorized social support interventions identified by the review as *Survivor focused*, *advocate/case management interventions (15 studies)*, survivor focused, advocate/case management interventions with a psychotherapy component (3 studies), *community-focused*, *social support interventions (6 studies)*, *community-focused*, *social support interventions with a psychotherapy component (3 studies)*. Most of the studies, resulted in improvements in social support and/or mental health outcomes of survivors, with little evidence of their effect on IPV reduction or increase in healthcare utilization.

**Conclusion:**

There is good evidence of the effect of IPV interventions focused on improving access to social support through the use of advocates with strong linkages with community based structures and networks, on better mental health outcomes of survivors, there is a need for more robust/ high quality research to assess in what contexts and for whom, these interventions work better compared to other forms of IPV interventions.

## Introduction

The global prevalence of intimate partner violence (IPV) has been estimated at about 30% for women aged 15 and over [1]. We define IPV within this paper as ‘any acts of physical violence, sexual violence, stalking and psychological aggression (including coercive tactics) by a current or former intimate partner’ [2]. IPV affects men and women, and men or women can be perpetrators or survivors of violence. However, women are the most affected by IPV, and men tend to perpetrate violence more than women [3]. Survivors of violence are likely to first disclose experiences of intimate partner violence and expect informal support from a friend, family member, neighbour or other members of their social network, prior to seeking support from formal sources like health institutions and legal officers, however, the extent of disclosure differed with age, nature, ethnicity and gender [4].

IPV has been found to be associated with an increased risk of poor health, depressive symptoms, substance use, chronic disease, chronic mental illness and injury for both men and women [5]. Social support has been found to be an important factor for mediating, buffering and improving the outcomes of survivors of violence and improving mental health outcomes[6]. Conversely, social isolation and lack of social support have been found to be linked with poor health outcomes for survivors of violence. Liang et al [6] discussed the importance, perception of the abuse by the IPV survivor plays on their decision to ask for help and support. They mentioned how cultural factors including stigma and shame around disclosing IPV, perception of the incident as a personal problem and awareness of resources available, play a determining factor on types of resources accessed, especially for IPV survivors with a migrant background or of a low socioeconomic status. IPV survivors who perceive the abuse to be a personal problem were more likely to use placating and avoidant strategies before seeking external support [6].

In this study, we make use of Shumaker and Brownell’s definition of social support, and define it as any provision of assistance, which may be financial or emotional, that is recognized by both the beneficiary and provider as advantageous to the beneficiary’s welfare. ‘[7]. IPV interventions that involve the use of social support, have the potential to improve the health seeking behaviour, access to resources and mental health outcomes of IPV survivors. Commonly cited types of social support interventions include but are not limited to the use of peer support, family support and the use of ‘remote interventions like the use of internet or telephones as sources of social support from trained counsellors, as well as information about resources’ [8]. Goodman and Smyth [9] discussed the importance of using a ‘network oriented’ approach to provision of domestic violence services that takes into account the value of informal support, from social network members of IPV survivors, as this would promote the well-being of the survivor and sustain some of the benefits of the intervention over time. Given the existing gap in evidence on the effect of different IPV interventions on social support and/ mental health outcomes of IPV survivors, this study aimed to address the evidence gap, by assessing the effects of these different IPV interventions, and network oriented approaches on improving access to social support and improved mental health outcomes for IPV survivors. This is of added benefit, as access to social support improves the mental health outcome of survivors of violence. More evidence of different types of social support interventions targeted at different groups of people, that are effective in addressing mental health outcomes of survivors, are needed.

## Methods

The systematic review was developed according to the PRISMA (Preferred Reporting Items for Systematic reviews and Meta-analyses) guidelines. The methods used to screen the studies and define eligibility are described below:

### Eligibility criteria

Studies meeting the following criteria were included: Primary research (original articles excluding systematic reviews), targeted at IPV survivors, describing interventions focused on improving access to resources and mental health outcomes for IPV survivors. The interventions had to use a social support or network-oriented approach. There were no restrictions on gender, but most of the studies identified focused on female survivors of violence (See Table 1). We defined ‘IPV as physical, sexual and psychological abuse directed against a person, by a current or ex-partner’ [10].

**Table 1:** Summary of studies selected.

Title of study	Authors	Type of intervention	Year	Type of study design	Target population/ control/ number of participants	Intervention	Main findings	Grading (EPPHP)
**1**. The impact of community-based outreach on psychological distress and victim safety in women exposed to intimate partner abuse	De Prince et al	Community focused support intervention	2012	Randomized control trial	236 Female adult Intimate partner violence survivors (non-sexual IPV)	Use of a systems based advocate (Referral) compared to a community coordinated/based advocate (Outreach) using telephone calls	***There was a moderate reduction*** in **PTSD, Depression and Fear. PTSD** (Outreach condition d: 0.43 (0.11, 0.74), and Referral condition d 0.31 (– 0.08, 0.70). RCI of 8.77 was calculated based on a reliability sample, where Cronbach’s alpha for PTSD 0.93 (SD 11.96), **Depression** (Outreach, t(206) 3.61, p .0004, d 0.37 (0.06,0.69), and Referral, t(210) 2.26, p .02, d 0.30 (– 0.09, 0.69), RCI of 8.50 was calculated based on a reliability sample where Cronbach’s alpha for depression 0.90 (SD 9.76) and **Fear**, (Outreach condition, t(205) 6.92, p .0001, d 0.77 (0.45, 1.09), to report greater reductions in fear symptoms than the Referral condition, t(208) 3.18, p .0017, d 0.45 (0.06, 0.85). RCI of .89 was calculated based on a reliability sample, where Cronbach’s alpha for fear 0.89 (SD 0.97); for both intervention groups **from baseline to 12 months after intervention**, with the outreach group with slightly higher scores. Other secondary outcomes like **Service Utilization**, **Outreach group**: (19%- contacted a community service, and 30% a therapist at 12 months), **Referral group** (13%- contacted a community service, and 37% a therapist at 12 months) there were no significant differences between both groups for service utilization and continued or new experiences of physical, sexual and psychological aggression. The probability of having left an abusive relationship was higher for the outreach group (d, t(56.60) 2.07, p.04). No changes from baseline were reported.	**Strong**
**2**. Effect of an advocacy intervention on mental health in Chinese women survivors of intimate partner violence: A randomized controlled trial	Tiwari et al	Survivor focused, Advocacy/ case management intervention	2010	Randomized control trial	200 Female adult Chinese IPV survivors (18 years and above)	Use of an advocate, empowerment counselling and weekly telephone calls for 12 weeks. The control received usual community services including child care, health care and promotion and recreation programs	The intervention reduced **depression scores** in the intervention arm compared to the control (2.66 (95% CI, 0.26 to 5.06; P = .03) vs the control) but it was not statistically significant, an impact was however noticed in reduction in partner aggression (psychological), At 9 months post baseline, Control (−6.4 [95% CI, −7.8 to −5.0] and Intervention −8.9 [95% CI, −10.6 to −7.2]) and increase in social support, At 9 months, Control: 12.4 [95% CI, 10.5 to 14.3] and Intervention: 14.4 [95% CI, 12.7 to 16.1]). Participants in the intervention arm reported improved quality of relationships((93.8% vs 81.7%; between-group difference, 12.1% [95% CI, 2.1% to 22.0%]; P = .02) and settling of disagreements with their partners (Intervention: 97.5% vs Control:84.1%; between-group difference, 13.4% [95% CI, 4.7% to 22.0%]; P = .001).	**Strong**
**3**. Findings from the SASA! Study: a cluster randomized controlled trial to assess the impact of a community mobilization intervention to prevent violence against women and reduce HIV risk in Kampala, Uganda.	Abramsky et al	Community-focused social support intervention	2014	Pair-matched Cluster Randomized control trial	Male, females, aged 18–49 four communities and 800 respondents per arm (100 men and 100 women per site)	The SASA! Activist Kit for Preventing Violence against Women and HIV is a community mobilization intervention that seeks to change community attitudes, norms and behaviours that result in gender inequality, violence and increased HIV vulnerability for women	There was a significant decrease in social acceptance of IPV among women (adjusted risk ratio 0.54, 95% confidence interval (CI) 0.38 to 0.79), and men (0.13, 95% CI 0.01 to 1.15) significantly greater acceptance that a woman can decline to have sex among women (1.28, 95% CI 1.07 to 1.52), and men (1.31, 95% CI 1.00 to 1.70); lower experience of physical IPV (last 12 months) among women, and lower levels of experience of sexual IPV (last 12 months) (0.76, 95% CI 0.33 to 1.72), Women experiencing violence in intervention communities were more likely to receive supportive community responses (2.11, 0.52 to 8.59). Reported multiple sexual partners (concurrent sexual partners) by men was significantly lower in intervention communities compared to control (0.57, 95% CI 0.36 to 0.91). Differences in past year physical/sexual IPV between intervention and control communities: Rates of sexual IPV increased in control communities, no difference in Intervention communities, and was statistically non-significant. Past year experience of physical IPV was substantially lower among intervention women compared to control women (0.48, 0.16 to 1.39), but was statistically non-significant. No mental health outcomes were reported	**Moderate**
**4**. An advocacy intervention program for women with abusive partners: initial evaluation.	Sullivan et al	Survivor focused Advocacy/case management intervention	1992	Randomized control trial	141 Adult female IPV survivors (battered women 18 years and above) who had spent a night at the shelter	Advocate worked with women for 10–12 weeks, Control group received usual services	There was a significant minimal reduction in depression, fear, anxiety, locus of control, self-efficacy, emotional attachment to assailant, physical and psychological abuse, social support and overall quality of life with a MANOVA test [F_(9,121)_ = 1.90, p = .058] across both groups but quality of life [F_(2,131)_ = 4.10, p < .05] and social support [F_(2,131)_ = 11.71, p < .05] significantly increased in the intervention group. Quality of life scores also increased between both groups, both groups (Control (51% physical violence,56% psychological violence) and intervention (40% physical abuse and 42% psychological violence) experienced abuse post-shelter, the difference was statistically non-significant	**Moderate**
**5**. An interpersonally based intervention for low-income pregnant women with intimate partner violence: A pilot study	Ziotnick et al	Survivor focused Advocacy/case management intervention with a psychotherapy component)	2011	Randomized control trial	54 Adult female IPV survivors who were pregnant between the ages of 18 and 40 years of age	Interpersonal therapy focused on improving social support, consisting of four 60-min individual sessions over a 4-week period before delivery and followed by one 60-min individual “booster” session within 2 weeks of delivery.	There was no significant reduction of the likelihood of a major depressive episode F(1, 44) = 1.73, p = 0.19 or PTSD F(1, 44) = 1.51, p = 0.23 between the control and intervention group postpartum (2 weeks and 3 months postpartum). However there were *moderate effects* for reducing symptoms of PTSD F(1, 44) = 7.50, p = 0.009 and Depression F(1, 44) = 3.29, p = 0.08 during pregnancy. The overall group effect across the two time periods was significant for the Depression and PTSD outcomes. Abuse victimization scores between the control and intervention group were not different or significant.	**Strong**
**6**. Effect of an in-clinic IPV advocate intervention to increase help seeking, reduce violence, and improve well-being.	Coker et al	Survivor focused Advocacy/case management intervention	2012	Quasi experimental study with randomization	Female adult IPV survivors, with experiences of IPV, in the 2 years prior to the study, and children between the ages of 4 and 6	The MEP intervention provides psychoeducation about violence and its effects on women and children, identifies and assists with advocacy needs, and teaches important skills for promoting good mental health, while working with advocates	There was an increase in use of support services (legal and social support) among the intervention group in the study compared to the control group (p = .003) and more likely in the first 6 months. There was also a greater decline of IPV among the research participants in the intervention group compared to the control group. IPV scores in the intervention clinics reduced over time relative to the control arm Scores for depressive symptoms and suicidal ideation were significantly lower over time for IPV+ women in the intervention clinics relative to the usual care arms. There were no differences between both groups in self-perceived physical health	**Moderate**
**7**. The process through which an advocacy intervention resulted in positive change for battered women over time.	Bybee et al	Survivor focused Advocacy/case management intervention	2002	Randomized control trial	Adult female IPV survivors	Advocacy/ Social support intervention, mediating and intervention effects assessed at 12 and 24 months follow up	The presence of social support and resources were found to be significant mediators of ‘quality of life’ scores in the ‘control’ and ‘intervention’ groups. Social support had more influence on outcomes (as a mediating factor) than access to resources for the research participants (p = 0.003). However this effect was not sustained over 24 months. At this time, social support played less of an influential role in outcomes, and access to resources had a significant effect. Women in the intervention arm (who worked with advocates) had positive outcomes sustained up to 24 months (quality of life, social support, access to resources and decline in experiences of repeated abuse).	**Moderate**
**8**. The Framing Safety Project: photographs and narratives by battered women.	Lisa Frohman	Community-focused social support intervention	2005	Ethnographic Qualitative study	Adult migrant female IPV survivors,	The Framing Safety Project is a therapeutic tool, a community education and action strategy, participatory action research for women to explore their experiences living in and extricating themselves from a battering relationship, and a means of informing others about the realities of these experiences, using photography.	The study reported an increased feeling of empowerment among women who participated in the project. The fact the intervention occurred within a support group context, seemed to make a difference for the participants, as this was a source of support and confidence. They were more willing to share their experiences of IPV with a public audience (using an exhibition) and be advocates for IPV prevention.	**Moderate**
**9**. Project WINGS (Women Initiating New Goals of Safety): A randomised controlled trial of a screening, brief intervention and referral to treatment (SBIRT) service to identify and address intimate partner violence victimisation among substance-using women receiving community supervision.	Gilbert et al	Survivor focused Advocacy/ case management intervention	2015	Randomized control trial	191 Adult women, with an intimate relationship with a history of substance abuse in the last 6 months	The WINGS intervention is guided by Social Cognitive Theory, it enables substance-using women to identify and disclose IPV, provide feedback on their risks for IPV, develop self-efficacy to protect themselves from IPV, raise awareness of drug-related triggers for IPV, develop safety plans considering substance-related risks for IPV and enhance social supports and linkages to IPV services, while working with case managers.	Disclosure rates were similar for the intervention-(Computerised:77.7%) and control arm (Case manager 77.3%), There were no significant differences between both arms in access to social support, reduction in drug use and utilization of IPV services. In both arms, there were improved scores across all outcomes: Received IPV services: Case Manager, Baseline (N = 148): 3 (4.0%), 3 months (N = 130) 11 (16.2%), OR IRR 7.72*(p<0.05), CI[95%] = [1.51, 39.45] Difference of change between case manager and computer 0.51 [0.07, 3.92], Computer: Baseline: 6 (8.3%), 3 months: 12 (19.4%) OR: 3.94*(p<0.05) CI[95%] [1.01, 15.38].	**Moderate**
10. Responding Effectively to Women Experiencing Severe Abuse: Identifying Key Components of a British Advocacy Intervention	Howarth and Robinson	Survivor focused Advocacy/case management intervention	2016	Retrospective quantitative study (Interrupted time series)	Adult domestic violence survivors who used an Independent domestic violence advisor, 2,427 (Time 1), 1,167 (Time 2)	The IDVA role was established in Britain in 2005, specifically to provide advice and support to survivors deemed to be at high risk of harm from further abuse. The IDVA model of intervention is designed to be delivered from the point of crisis over a relatively short period of time and is focused on addressing immediate risks to safety and barriers to service utilization, before referring victims to other services	Women who had more interaction with the advocates were less likely to experience abuse, reported significant reductions in the occurrence of all forms of abuse, **sexual abuse**: % reporting sexual abuse (13.1 vs. 14.0) T1, 24.5 T2, 5.0**(p<0.01), % reporting perpetrator’s jealous/controlling behaviour (5.4 vs. 26.9) T1, 87.2 t2, 26.9**(p<0.01) % reporting any form of severe abuse T1, 79.2 T2, 19.2**(p<0.01) % reporting any escalation in severity or frequency of abuse T1, 60.6 T2, 4.2**(p<0.01) and importantly most felt to some degree safer. 1.4% of women reported to IDVAs that they felt significantly safer and 23.5% felt somewhat safer. Although women living separately from the abuser at Time 1 were more likely to report positive outcomes at Time 2	**Weak**
11. Secondary Prevention of Intimate Partner Violence A Randomized Controlled Trial	McFarlane et al	Survivor focused Advocacy / case management intervention	2006	Randomized controlled trial	Pregnant IPV survivors with children 5 years or younger	The intervention tested the added benefit of a nurse case management, in addition to the use of an abuse assessment and a referral card, based on the ‘March of Dimes’ protocol	There were no significant differences between groups across all outcomes, i.e. No added benefit of using a nurse case manager. Two years following treatment, both treatment groups of women reported significantly (p < .001) reduced experiences of abuse threats, assaults, harassment at work and risks of homicide but there were no significant differences between groups. Compared to baseline, both groups of women adopted significantly (p < .001) more safety behaviours by 24 months (however, community resource use declined significantly (p < .001) for both groups. There were no significant differences between groups.	**Moderate**
12. Preventing Abuse to Pregnant Women: Implementation of a 'Mentor Mother' Advocacy Model	McFarlane et Wilst	Survivor focused Advocacy/case management intervention	1997	Pre and post evaluation	100 Pregnant IPV survivors leaving shelters	The intervention focused on the effectiveness of the mentor mother model in improving effective referrals	The programme was effective in improving the number of referrals among the women who used the advocacy services, and was reported to be effective ‘in offering social support, education and assistance with needed community resources’.	**Weak**
13. A Trial of Telephone Support Services to Prevent Further Intimate Partner Violence	Stevens et al	Survivor focused Advocacy/ case management intervention	2015	Randomized control trial	300 Adult IPV survivors with partial or full custody of a paediatric patient at the emergency department	The advocacy intervention consisted of empowerment and telephone social support, based on the Dutton’s empowerment model and Cohen’s Social Support Theory. The former component was provided at the beginning of the intervention and took about 30 minutes. It included protection and enhanced choice-making and problem-solving skills. The latter was provided via 12 scheduled weekly telephone calls and 24-hour access to a hotline. Women in the control group received the usual community services provided by the community centre including health, social, educational, and recreational services.	The study found higher perceived levels of social support at baseline and 3 months was mildly related to reduced reporting of experiences of violence (CAS scores) and, and there were significant reductions in IPV victimization across all groups, no differences between groups or other outcomes were noted.	**Strong**
14. The process, outcomes, and challenges of feasibility studies conducted in partnership with stakeholders: a health intervention for women survivors of intimate partner violence	Wuest et al	Community-focused social support interventions	2015	Pre-post study	52 Adult IPV survivors, who are living apart from their abusive partners for 3 months	The protocol is used during 12 to 14 individual sessions with an interventionist over a six-month period. The six components of the iHEAL are addressed in three phases. In getting in sync, interventionists and women begin to build mutual trust by discussing the woman’s priorities, the survival context, and the nature of the iHEAL and the SCLI theory, as planning the order of their work on components. In working together, for each component, women are supported to frame their personal experiences of intrusion in light of what is known about other survivors’ intrusion experiences, using paper-based tools or exercises developed for this purpose.	Preliminary effects show improvement in the quality of life and mental health of the IPV survivors. There was also a reduction in symptoms of depression and PTSD	**Moderate**
15. Community-based PTSD treatment for ethnically diverse women who experienced intimate partner violence: a feasibility study.	Kelly et al	Community-focused social support intervention with a psychotherapy component	2014	Pre and post study	12 Adult IPV Spanish immigrant women with symptoms of PTSD	The initial intervention consisted of 6–10-session weekly psychotherapy groups, using a synthesis of supportive psychotherapy, including psycho-education and self-care strategies. Specific topics included PTSD 101; relationships with self, children, and others; sleep hygiene, mindful eating, the relaxation response, and exercise; faith and family. The intervention was conducted by two advanced practice psychiatric nurses (PMHCNS and PMHNP), with experience in treating survivors of IPV with PTSD.	Symptoms of PTSD decreased at 6 months post-intervention, from 59.00 to 44.13, respectively (n = 15; p = 0.003). Symptoms of MDD decreased significantly at all post-intervention time-points. The only statistically significant difference in self rating of general health was from baseline (3.06) to 6-month follow-up (2.63; p = 0.048). Self-reported mental health-related QoL also improved significantly at all time-points, with a nearly 50% (p = 0.013) and 66% improvement (p = 0.003) at 3- and 6-month follow-up, respectively. Self-efficacy scores improved at all time-points, reaching statistical significance (p = 0.020) at 6-month follow-up	**Moderate**
16. The effect of an evidence-based intervention on women's exposure to intimate partner violence	Miller et al	Survivor focused support Advocacy/ case management intervention with a Psychotherapy-based	2014	Randomized control trial	120 Adult IPV survivors who have had experiences in the last 2 years	The current study aimed to examine the effectiveness of the partner evidence based intervention for adult women, the Moms’ Empowerment Program (MEP; Graham-Bermann, 2000), on women’s violence victimization. This is a 10-session group intervention program for women who have experienced IPV in the past 2 years. The MEP provides psychoeducation about violence and its effects on women and children, identifies and assists with advocacy needs, and teaches important skills for promoting good mental health (e.g., processing traumatic events, relaxation techniques).	At the 6- to 8-month follow-up, women in the comparison group reported an average of 12 (SD 24) events of physical violence, sexual coercion, and violence-related injury that had occurred since the baseline interview, whereas those women in the intervention reported an average of 3 events. (SD 10). There was no significant difference between groups. There was also a significant decrease in IPV over time for all women, regardless of treatment status (0.04, p .001). However, women in the intervention group, had higher reductions in IPV compared to women in the control group.	**Weak**
17. Effects of an intervention program for female victims of intimate partner violence on psychological symptoms and perceived social support	Hansen et al	Survivor focused Advocacy/case management intervention with a Psychotherapy component	2014	Pre-post test	212 Adult female IPV survivors	The intervention program in the present study was based on the TRG method and the traumatic treatment approach presented by Mendelsohn et al. (2011) and Herman (1997), the intervention program in this study followed a three-phase stepwise treatment approach, 1) Stabilisation program, 2) Treatment program, 3: the follow-up program respectively.	Stabilization program phase: Participants who completed this phase had significant reductions in PTSD, depressive symptoms and anxiety symptoms (from baseline to post-intervention). They also had a significant increase in levels of perceived social support from baseline to completion. Participants who completed the treatment program experienced significant reductions in PTSD depressive symptoms and anxiety symptoms (from baseline to completion of the treatment program). There was no significant difference in levels of perceived social support.	**Weak**
18. Differential therapeutic outcomes of community-based group interventions for women and children exposed to intimate partner violence.	Paula T. McWhirter	Community-focused social support intervention with a psychotherapy component	2011	Randomized control trial	IPV survivors with children, aged between 6 and 12 years	The study involves an evaluation of an emotion focused group interventions versus a goal focused intervention for IPV	These results suggest that participants in both groups demonstrated decreased family conflict and improved quality of social support; however, significantly greater reduction in family conflict was reported among women who participated in the goal-oriented intervention compared to those participated in the emotion-focused intervention, and significantly greater increases in social support was reported among women who participated in the emotion-focused intervention compared to those who participated in the goal-oriented condition. Women in both groups reported decreases in depression and increases in family bonding and self-efficacy, as well as reduction in alcohol use	**Moderate**
19. Proyecto Interconexiones: A Pilot Test of a Community-Based Depression Care Program for Latina Violence Survivors	Nicoladis et al	Community-focused social support intervention with a psychotherapy component	2013	Pre-post study	10 Spanish speaking IPV survivors	The promotora served as the care manager, helping women gain access to or navigate the healthcare system and providing case management services as needed over a 6- month period. The Project Coordinator served as the promotora’s assistant and co-facilitator, the intervention also consisted of therapy sessions from the beginning of the program focusing on domestic violence.	There was a significant reduction in depression scores from 18.8 to 7.4 (p = 0.002). They also reported increases in self-esteem and depression self-efficacy, that were statistically significant. There was reported increase in the level of acceptance of antidepressants	**Moderate**
20. Support by trained mentor mothers for abused women: a promising intervention in primary care	Prosman et al	Survivor focused Advocacy/case management intervention	2014	Pre and post	63 Adult IPV survivors with children up to 18 years	A comprehensive education programme for mentors was developed for effective support. The mentor support consisted of 16 weekly home visits by a trained mentor mother. Four different protocols were developed, which described the interventions during the 16 weeks in detail. This programme focuses on establishing a friendly supportive relationship with the abused woman, in guiding her in dealing with IPV, depressive symptoms and in strengthening social and parenting support and acceptance of professional mental health care, both for mother and child	Among the research participants, experience of partner violence reduced, as well as symptoms of depression. which decreased by 35%, from baseline 53.3 (SD 13.7) to 34.8 (SD 11.5), 95% CI: 14.4–22.6 (P ≤ 0.001), after the intervention. The social support scores increased with 15% significantly from baseline	**Weak**
21. Effects of a social support intervention on health outcomes in residents of a domestic violence shelter: A pilot study	Constantino et al	Survivor focused Advocacy/ case management intervention	2005	Randomized control trial	24 Adult IPV survivors with children	The interventions was planned as an eight-week, once every week 90-minute program led by a trained nurse. The intervention was designed to provide resources to women and included information on resources, time to access resources when available, and environment to chat with counsellor and friends. The control group received the usual shelter services	The intervention group had greater improvement (p = .013) in psychological distress symptoms and greater improvement in perceived availability of social support (p = .016) than the control group. The intervention group showed less health care utilization (p = .032) than the control group. Social support interventions for women in shelters are effective in improving health outcomes.	**Weak**
22. Comparing Online with Face-to-Face HELPP Intervention in Women Experiencing Intimate Partner Violence	Constantino et al	Survivor focused Advocacy/case management intervention	2015	Randomized control trial	32 Adult female IPV survivors	The Online and ‘face to face’ intervention consisted of six modules. The online module was delivered by email and the ‘face to face’ intervention was given in person. Each HELPP module presents a title, learning objectives, educational content, assignments, and prompts for reflection and self-evaluation. The titles for each module are (1) Personal Thoughts, Emotions, and Behaviour; (2) Interpersonal Relationships and Healing in Telling; (3) Health in HELPP; (4) Education on Safety in HELPP; (5) Legal Matters in HELPP; and (6)Community and the A-B-Cs of Empowerment.	The HELPP intervention (1) decreased anxiety, depression, anger, and (2) increased personal and social support in the ONL group. The HELPP information and intervention was shown to be feasible, acceptable, and effective among IPV survivors compared with participants in the WLC group.	**Moderate**
23. Mothers’ AdvocateS In the Community (MOSAIC)-non-professional mentor support to reduce intimate partner violence and depression in mothers: a cluster randomised trial in primary care	Taft et al	Survivor focused Advocacy/ case management intervention	2011	Cluster randomized control trial	Pregnant IPV women with children 5 years or younger	The MOSAIC (MOtherS’ Advocates In the Community) model trialled in the current study combined evidence for the benefits of social support, advocacy, and antenatal mentoring to reduce partner violence and improve women’s mental and physical health. We located the study in primary care to contribute to the limited evidence about effective referral and intervention strategies in this setting and because mothers experiencing IPV are more prevalent in these populations	There was an observed reduction in the level of partner violence (weak evidence) and depression among the mentored group. There was weak evidence of improved general health and well-being among the mentored group	**Moderate**
24. Domestic Violence Enhanced Perinatal Home Visits The DOVE Randomized Clinical Trial	Sharps et al	Survivor focused Advocacy/case management intervention	2016	Randomized control trial	239 Adult pregnant IPV survivors	The goal of the study was to test whether integrating a structured IPV intervention, DOVE, into a home visiting program, regardless of model, would in-crease the safety in perinatal women experiencing violence. All home visiting programs have an essential component of community health nursing practice to improve health outcomes for families (parents or children), but screening and intervening with women experiencing violence have not historically been integrated into the different models.	The intervention resulted in a significant decrease in experience of IPV in the intervention arm (compared to the control group).	**Strong**
25. Reducing violence using community-based advocacy for women with abusive partners.	Sullivan et al	Survivor focused Advocacy/case management intervention	1999	Randomized control trial	278 Adult IPV survivors	Advocates were trained female undergraduates and required to provide support to IPV survivors 4-6hrs/week	Research participants involved in the intervention reported a decline in physical and psychological violence, as well as a decline in depression levels and an increase in perceived quality of life and social support. Compared over time, decline in level of psychological abuse and increase in quality of life, was significant for the intervention group.	**Strong**
26. SASA: Findings from the qualitative study	Kyegombe et al	Community-focused social support intervention	2014	Qualitative study	40 Community members	The SASA! Intervention is focused at promoting community-level responses to gender based violence, by creating a phased change process that takes communities through a structured programme of discovery, critical reflection, and skills building	The SASA intervention changed the perception of violence and gender roles among men and women in the communities targeted by the intervention. Due to the intervention, there was reduced acceptability of intimate partner violence and more support for GBV survivors in the community. Also, by addressing underlying gender roles. It encouraged women to disclose experiences of violence and for men to openly discourage intimate partner violence.	**Strong**
27. Qualitative Study of an operations research project to engage abused women, health providers and communities in responding to gender based violence in Vietnam	Schuler et al	Community-focused social support intervention	2011	Qualitative research: Action research project	146 Adult Married women (IPV survivors), Married men, Key informants and health service providers	The project design was also informed by a theoretical model of GBV described by Heise(1998), who encourages the widespread adoption of an integrated, ecological framework for understanding the origins of GBV. This approach to abuse conceptualizes GBV as a multifaceted phenomenon reflecting an interplay among personal, situational, and sociocultural factors. The project design emphasizes the creation of a comprehensive network for behaviour change communication and support to abused women through a variety of institutions.	The community members involved in the study reported an increased perception of their right and obligation to intervene in GBV cases in their community. There was also an increase in community support for GBV survivors and the survivors reported increased support seeking behaviour and increased awareness of their rights as individuals (against GBV) and as a group.	**Moderate**

Studies had to address the following outcomes: intimate partner violence, social support, mental health outcomes and quality of life. Other outcomes that were also included were those associated with access to resources, utilisation of health services, and safety-promoting behaviours, if they were assessed in addition to the outcomes mentioned earlier. No restrictions were placed on study design or language, to allow for inclusion of all relevant studies.

### Information sources

Between May and July 2017, we conducted a search across 5 databases: Medline, Embase, Web of Science, Cochrane and PROQUEST, for studies published between 1980 and 2017. We decided to include studies from the 1980’s because some of the pioneering publications on the use of advocacy and social support, for example, Sullivan et al’s work were published in the late 80’s and early 1990’s and we wanted our review to include some of these publications. Even though the review eventually included only primary studies, we included studies from COCHRANE to allow us to identify additional articles. We did not conduct a separate search for grey literature, as the PROQUEST database also included scholarly journals, newspapers, reports, working papers, and datasets along with e-books. Retrieved references were imported to Endnote and Mendeley and were then transferred to a systematic review software called Co-evidence [11]. In January 2019, another search was done to update and ensure new articles or information could be included in the review. Table 1 provides an overview and summary of the studies selected, as well as the evidence ranking of the studies.

### Search strategy

The search strategy was developed in collaboration with a librarian, as well as a review of other existing systematic reviews on IPV or social support interventions. Search terms combined MeSH terms, and specific terms related to IPV and were adapted to each of the databases searched. This is presented in Table 2.

**Table 2:** Search terms and articles retrieved from each database.

Name of database	Last date accessed	Search terms	Total retrieved
PubMed	19 May 2017	Search (((('domestic violence'/exp OR 'domestic violence' OR 'family violence'/exp OR 'family violence' OR 'partner violence'/exp OR 'partner violence' OR 'battered woman'/exp OR 'battered woman' OR 'spouse abuse'/exp OR 'spouse abuse' OR 'spousal abuse' OR 'abused mothers' OR 'dating violence'/exp OR 'dating violence' OR 'date rape'/exp OR 'date rape' OR (battered OR batter OR abuses OR abusive OR shelters OR violent OR shelter OR 'violence'/exp OR 'violence' OR 'violence'/exp OR violence OR 'abuse'/exp OR 'abuse' OR 'abuse'/exp OR abuse OR abused OR 'battering'/exp OR 'battering' OR 'battering'/exp OR battering OR victimization OR 'rape'/exp OR 'rape' OR 'rape'/exp OR rape AND ('intra-family' OR 'intra family' OR marital OR spouse* OR spousal OR wife OR wives OR husband OR husbands OR couples OR partners OR partner OR 'male to female' OR 'mother'/exp OR 'mother' OR 'mother'/exp OR mother OR 'mothers'/exp OR 'mothers' OR 'mothers'/exp OR mothers OR 'pregnant women'/exp OR 'pregnant women')) OR 'ipv')) AND (((((((community network[MeSH Terms]) OR social network[MeSH Terms]) OR psychosocial support system[MeSH Terms]) OR social networking[MeSH Terms]) OR care network, community[MeSH Terms]) OR social network[Title/Abstract]) OR social support[Text Word])) AND (((((((((((health promotion[MeSH Terms]) OR health education[MeSH Terms]) OR health education, community[MeSH Terms]) OR primary prevention[MeSH Terms]) OR public health[MeSH Terms]) OR preventive health service[MeSH Terms]) OR preventive medicine[MeSH Terms]) OR health communication[MeSH Terms]) OR intervention studies[MeSH Terms]) OR pilot project[MeSH Terms]) OR health intervention[Title/Abstract])) Sort by: [pubsolr12]	1788
Embase	19 May 2017	domestic violence'/exp OR 'domestic violence' OR 'family violence'/exp OR 'family violence' OR 'battered woman'/exp OR 'battered woman' OR 'spouse abuse'/exp OR 'spouse abuse' OR 'spousal abuse' OR 'abused mothers' OR 'dating violence'/exp OR 'dating violence' OR 'date rape'/exp OR 'date rape' OR (battered OR batter OR abuses OR abusive OR shelters OR violent OR shelter OR 'violence' OR 'violence'/exp OR violence OR 'abuse' OR 'abuse'/exp OR abuse OR abused OR 'battering' OR 'battering'/exp OR battering OR 'victimization'/exp OR victimization OR 'rape' OR 'rape'/exp OR rape AND ('intra-family' OR 'intra family' OR marital OR spouse* OR spousal OR 'wife'/exp OR wife OR wives OR 'husband'/exp OR husband OR husbands OR 'couples'/exp OR couples OR partners OR 'partner'/exp OR partner OR 'male to female' OR 'mother' OR 'mother'/exp OR mother OR 'mothers' OR 'mothers'/exp OR mothers OR 'pregnant women'/exp OR 'pregnant women')) OR 'partner violence'/exp OR 'partner violence' AND ('health education'/exp OR 'health program'/exp OR 'medical information'/exp OR 'preventive medicine'/exp OR 'intervention study'/exp OR 'pilot study'/exp OR 'public health service'/exp OR 'intervention study':ab,ti) AND ('social network'/exp OR 'community support'/exp OR 'psychosocial care'/exp OR 'social support'/exp OR 'social network':ab,ti OR 'social support':ab,ti)	423
Web of science	28 July 2017	TS = (domestic near/3 violence OR spous* near/3 abuse OR dat* near/3 violence OR dat* near/3 rape OR wife near/2 batter* OR partner near/3 violence)	186
		AND	
		TS = (social near/3 net* OR social near/3 support OR commun* near/3 support OR commun* near/3 net* OR psychosocial* near/3 support OR care near/3 network)	
		AND	
		TS = (health near/3 promot* OR health near/3 educat* OR health near/3 interven* OR health near/3 prevent* OR interven* near/3 project OR pilot near/3 project OR intervene* near/3 stud*)	
Cochrane	29 July 2017	'domestic violence' or 'family violence' or 'battered woman' or 'spouse abuse’ or 'spousal abuse' or 'dating violence' or 'date rape' or battering or victimization or rape or ' intimate partner violence' or ‘sex offense*’ or ‘sexual violence’ or ‘sexual assault*’ or ‘sexual violation’ or ((partner or spous* or domestic or dating or couple or marital or wife or wive*) near/2 (abuse or aggression or violence or assault or maltreatment)) or rape or ‘battered wom?n’ or (gender* near/2 violence) or (violence near/2 wom?n)	169
		AND	
		(Link* near/2 (care or healthcare or service* or therap* or treatment*)):ti,ab or (access* near/2 (care or healthcare or service* or therap* or treatment*)):ti,ab or (utiliz* near/2 (care or healthcare or service* or therap* or treatment*)):ti,ab or (seek* near/2 (care or healthcare or service* or therap* or treatment*)):ti,ab or 'health intervention' or 'pilot study' or 'public health'	
		AND	
		[mh ^"Social Support"] or [mh "community networks"] or "social network" or "social support" or "community support" or ("support system*" or "social support" or "community support" or "social network*" or "community network*" or "community involvement" or "family support" or "family involvement" or "family network" or "parental support" or "parental involvement" or "parental support" or assistance or encouragement or coping or "better cope" or "peer involvement" or "peer support" or "peer network*" or friends):ti,ab	
PROQUEST	29 July 2017	('domestic violence' OR 'family violence' OR 'battered woman' OR 'spouse abuse’ OR 'spousal abuse' OR 'dating violences' OR 'date rape' OR battering OR victimization OR rape OR ' intimate partner violences' OR ‘sex offense*’ OR ‘sexual violences’ OR ‘sexual assault*’ OR ‘sexual violation’ OR ((partner OR spous* OR domestic OR dating OR couple OR marital OR wife OR wive*) NEAR/2 (abuse OR aggression OR violence OR assault OR maltreatment)) OR rape OR ‘battered wom?n’ OR (gender* NEAR/2 violence) OR (violence NEAR/2 wom?n))	1304
		AND	
		((LNK* NEAR/2 (care OR healthcare OR service* OR therap* OR treatment*)):AB,TI OR (access* NEAR/2 (care OR healthcare OR service* OR therap* OR treatment*)):AB,TI OR (utiliz* NEAR/2 (care OR healthcare OR service* OR therap* OR treatment*)):AB,TI OR (seek* NEAR/2 (care OR healthcare OR service* OR therap* OR treatment*)):AB,TI)	
		AND	
		((“support system*” OR “social support” OR “community support” OR “social network*” OR “community network*” OR “community involvement” OR “family support” OR “family involvement” OR “family network” OR “parental support” OR “parental involvement” OR “parental support” OR assistance OR encouragement OR coping OR “better cope” OR “peer involvement” OR “peer support” OR “peer network*” OR friends):AB,TI)	

### Study selection

Inclusion of retrieved studies and their eligibility were independently assessed by two reviewers, EO and SH, in a two-step process. First, the authors independently screened all study titles and abstracts using Co-evidence (the systematic review software), which notified each author of conflicts. When a conflict was identified, articles were again independently reviewed, and discordance was resolved through discussion, using the systematic review protocol as a guide. The same process was also used for the full text-screening phase of the study. While this process lengthened the screening process, it allowed for transparency and made it possible for both reviewers to continually reference the study protocol and ensure that the study objectives were adhered to, through the review process.

### Data extraction

A standardized data collection form was developed by EO and SH, adapted from the Cochrane data collection grid. EO extracted all the data from the studies, SH and RB reviewed the data and it was agreed that OD would provide input if there was any disagreement about the data extracted.

### Risk of bias

The quality and risk of bias in the studies were independently assessed by EO and SH, using the appropriate quality assessment tool. As the studies selected included quantitative and qualitative studies, there was an agreement to assess quantitative and qualitative studies separately. Quantitative studies were assessed using the Quality Assessment Tool for quantitative studies developed by the Effective Public Health Practice Project, see Table 3 for an overview of the components of this tool [12]. This tool had been used in another systematic review focused on interventions [13]. Qualitative studies were assessed, using the Critical Appraisal Skills Programme (CASP) Qualitative Research Checklist [14], the main components focused on assessing the methodological limitations, coherence, adequacy of data and relevance of research. See Table 4 for an overview.

**Table 3:** Ranking criteria used for quantitative studies: Effective public health practice project: Quality assessment tool for quantitative studies. [12].

	STUDY COMPONENT	STRONG	MODERATE	WEAK
**A**	**SELECTION BIAS**	1	2	3
**B**	**STUDY DESIGN**	1	2	3
**C**	**CONFOUNDERS**	1	2	3
**D**	**BLINDING**	1	2	3
**E**	**DATA COLLECTION METHOD**	1	2	3
**F**	**WITHDRAWALS AND DROPOUTS**	1	2	3
**G**	**INTERVENTION INTEGRITY**	1	2	3
**H**	**ANALYSIS APPROPIRATE TO QUESTION**	1	2	3
	**GLOBAL RATING**	**STRONG (***No weak rating)*	**MODERATE (***One weak rating)*	**WEAK (***Two or more weak rating)*

**Table 4:** CASP qualitative studies checklist.

Section	Questions	Yes	Can’t tell	No
**Section A: Are the results valid**	Was there a clear statement of the aims of the research?			
	Is a qualitative methodology appropriate?			
	Was the research design appropriate to address the aims of the research?			
	Was the recruitment strategy appropriate to the aims of the research?			
	Was the data collected in a way that addressed the research issue?			
	Has the relationship between researcher and participants been adequately considered?			
**Section B: What are the results**	Have ethical issues been taken into consideration?			
	Was the data analysis sufficiently rigorous?			
	Is there a clear statement of findings?			
**Section C: Will the results help locally**	How valuable is the research?			

## Results

### Information about studies selected

**Fig 1:**
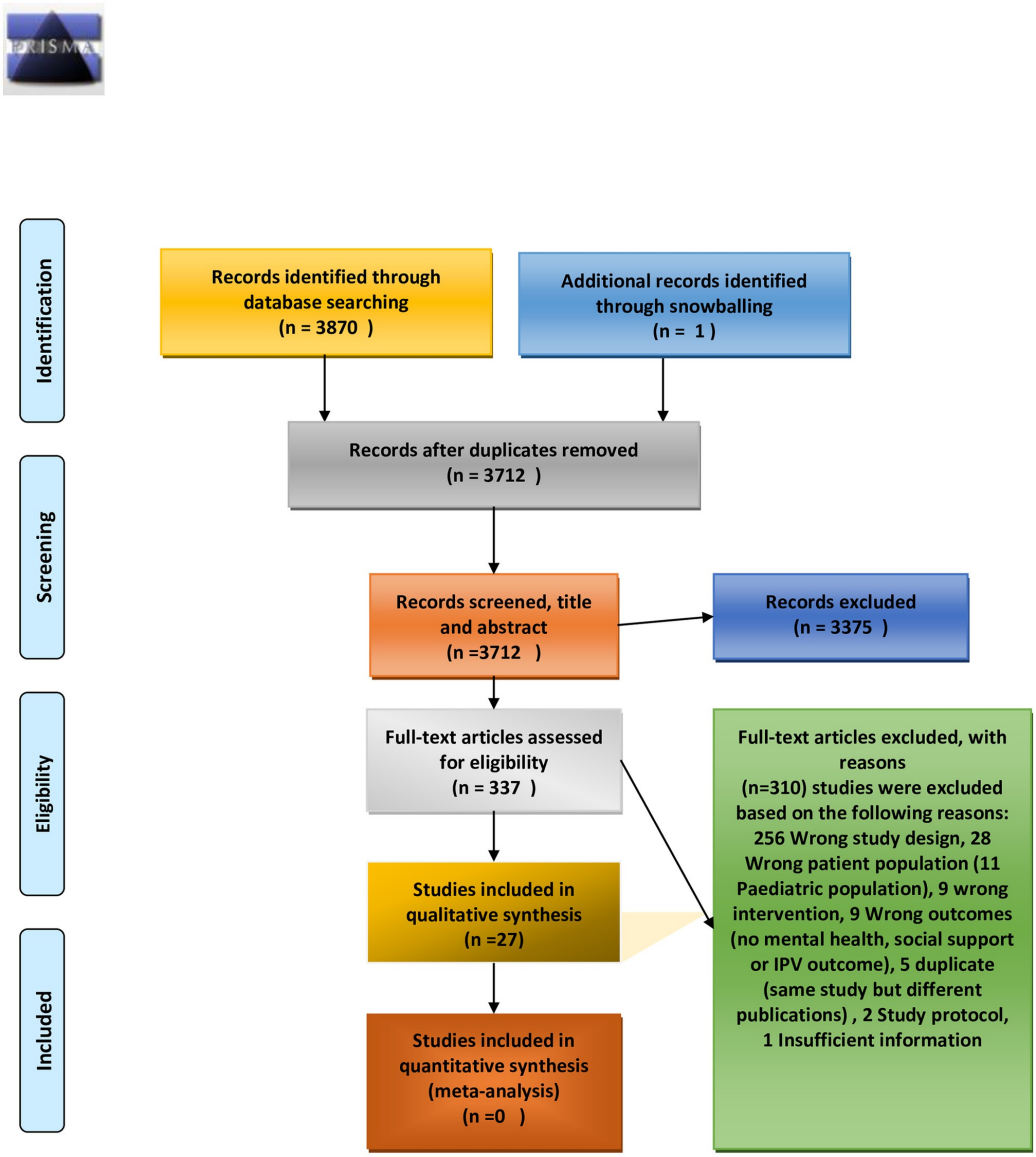
PRISMA flow diagram for the systematic review of IPV interventions focused on improving social support and mental health outcomes among IPV survivors. *From*: Moher D, Liberati A, Tetzlaff J, Altman DG, The PRISMA Group (2009). *P*referred *R*eporting *I*tems for *S*ystematic Reviews and *M*eta-*A*nalyses: The PRISMA Statement. PLoS Med 6(7): e1000097. doi: 10.1371/journal.pmed1000097 **For more information, visit** www.prisma-statement.org.

The initial search across the different databases retrieved 3712 articles, of which 3364 articles were irrelevant based on the screening criteria. 337 articles were assessed at the full text screening stage, and 27 articles selected to be part of the systematic review, the overview is presented in Fig 1

### Results/Key findings from the systematic review

The interventions were classified based on the methodology or type of social support provided to the survivors of violence. Most of the studies identified involved the use of an ‘advocate/ case manager’ or ‘interventionist’ (which referred to a nurse, psychologist or volunteer trained to administer the IPV intervention). The advocate was often responsible for offering the survivor information on resources and helping them identify safety strategies. The interventions usually consisted of weekly sessions or phone calls for a certain period of time. These interventions were mostly in the United States and from other countries like China, Canada, Denmark, Netherlands, Uganda and the United Kingdom. Other interventions involved the use of advocacy with an added psychotherapy component, and interventions that focused on community education, as well as empowerment of the IPV survivors. One of such community focused interventions used an empowerment model and encouraged survivors of violence to take photos of their safety strategies. These photos were used to educate the community about the consequences of intimate partner violence and advocate for community support to prevent intimate partner violence and encourage access to services. In our paper, the term ‘community focused’ included interventions targeted at the community which used participatory and non-participatory methods in the design and implementation of the programmes. The interventions identified in this systematic review had different target groups, pregnant women, survivors of violence resident in shelters, community members and IPV survivors, substance abusing women, and women with small children.

### Types of social support interventions for intimate partner violence survivor

#### Survivor- focused social support interventions

The interventions described below were all focused on providing social support and improving mental health outcomes for the survivors of violence, all of them involved the use of advocacy/case management approaches, through remote or ‘face to face’ methods. We also identified advocacy interventions with a strong therapeutic component, which we have discussed separately.

### Advocacy/ Case management interventions

These interventions involved the use of community-based advocacy interventions focused on individuals that were survivors of violence, these interventions were focused on assisting the survivors identify and access resources, supportive relationships and cope with the effects of intimate partner violence. Fifteen of the studies reviewed (11 RCTs, 2 pre-post evaluation, 1 retrospective study, 1 quasi-experimental study with randomization) described experiences with social support interventions that provided some sort of advocacy service in combination with community support for survivors of violence, on an individual level [15–29].

Advocacy interventions may include ‘helping abused women to access services, guiding them through the process of safety planning, and improving abused women’s physical or psychological health’ [30]. For the review, interventions grouped under this category included mentor-mother interventions (these interventions involved the training of IPV survivors who were mothers as counsellors and mentors, for other IPV survivors), and use of home-based or in-clinic advocates. Most of the studies reported a decrease in depression, fear, post-traumatic stress disorder, and increased access to social support for the IPV survivors included in the study.

In Tiwari et al’s study, where an advocacy intervention was compared to the usual community services, the reduction in depression and other mental outcomes, was not significant but the reduction in ‘partner aggression’ and increase in access to social support in the intervention arm was significant [15]. Two of the studies, an in-clinic advocacy intervention by Coker et al [23] and a home-based advocate intervention by Sharps et al [20] resulted in a significant reduction in the experience of intimate partner violence by the survivors (decrease in experience of IPV in the intervention arm compared to the control group). The two mentor mothers’ studies included in this review, showed an increase in uptake of support services and mental health services. Prosman et al’s study [18] specifically showed evidence that the mentor mother intervention led to a decrease of in experience of IPV (decreased Composite Abuse Scale (CAS) mean score by 37.7 (SD 25.7) after 16 weeks), as well as in depression scores. This study had a component that focused on uptake of therapy, which may have influenced the outcomes. Four of these studies compared ‘face to face’ case management/ advocacy services to remote modes of care and assessed the impact on social support and IPV. Gilbert et al’s study [24] compared online and case manager implemented screening, assessment, and referral to treatment intervention for IPV survivors who were substance abusing, the intervention was guided by social cognitive theory, and focused on short screening, an intervention and referral to treatment (SBIRT) model. There were no significant differences between both groups in terms of impact of the interventions, the study found both groups has an increase in access to social support, IPV self-efficacy (ability to protect themselves from IPV) and abstinence from substance use, irrespective of the type of intervention they received. McFarlane et al [26] assessed the differences between nurse case management and a referral card on reduction of violence and use of community resources among IPV survivors, and found no differences in outcome between both groups, but found compared to baseline, participants who received either intervention (nurse case management or referral card) had a significant reduction in experiences of violence (threats of abuse, assaults, risks of homicide and work harassment) between baseline and 24 months post-intervention. There were no significant differences in outcome for participants who were in the referral card or case management intervention arm. Other outcomes like improved safety behaviors and a reduction in the utilization of community resources were also found across both groups. Stevens et al’s [27] study focused on using telephone based support/referral services for IPV survivors compared to enhanced usual care (, the intervention was based on a social support and empowerment model. The study found no significant difference in outcomes between the intervention arm (telephone-based arm) and the control arm (enhanced usual care- community services provided by the community center including health, social, educational, and recreational services). Research participants reported a decrease in experiences of IPV across both groups, associated with ‘higher levels of social support’ at baseline and at 3 months post-intervention. However, the reduced levels of violence did not influence the capacity to obtain or utilize community resources among the research participants. Constantino et al’s [29] study compared an advocacy based intervention across different methods (online and face to face) and found the intervention reduced depression, anxiety and increased personal and social support among the online group compared to the control group. The intervention included a module that addressed interpersonal relationships, thoughts and emotions as well as access to referral services like legal aid. Another study by Constantino [28] involved a nurse led intervention focused on providing information on resources and services for IPV survivors living in a domestic violence shelter. The intervention was compared to usual care in the shelter. The intervention group had reduced psychological distress, increased levels of social support and reduced reporting of health care issues. Most of the studies we found in this category showed moderate levels of quality of evidence.

### Advocacy/Case management interventions with a psychotherapy component

3 of the studies (3 RCTs) [31–33] were focused on interventions that included specific types of psychotherapy, sometimes delivered remotely or through individual or group sessions. Zlotnick et al [31] described the use of interpersonal psychotherapy among pregnant women focused at improving social support among the survivors of violence during individual psychotherapy sessions. Though there was a moderate change in depression and PTSD scores (reduction) between the control and intervention groups at post-intake (5–6 weeks), this difference was not sustained at the post-partum period. Hansen et al [33] describes the use of psychotherapy using either the ‘Trauma Recovery Group’ (TRG) method developed by ‘a private Danish organization called ‘‘The Mothers’ Aid”‘ or regular trauma therapy for individual or groups of women who were survivors of IPV. The study reported significant changes in PTSD, depression and anxiety symptoms and increased levels of social support (high effect sizes); however, our assessment with the EPHPP grading revealed that the study design was weak. Miller et al’s [32] study shows the effect of a ‘mom empowerment programme’ focused on improving mental health outcomes and ability to access resources among IPV survivors participating in the programme, with resulting improvement in PTSD, depression and anxiety symptoms.

### Community-focused/ network social support interventions

These group of studies, distinct from the ones described above focused on community education and change, so the focus of the studies was not just the individual survivor of violence, but the community as a whole. 9 (3 RCTs, 3 pre-post evaluations, 3 qualitative research) of the studies we reviewed consisted of interventions described as being community-based [34–42]. The definitions of community-focused interventions used for classifying the studies followed the typology by McLeroy et al [43], which refers to interventions where:

- The setting of the intervention is the community
- The target population of the intervention is the community
- The intervention uses community members as a resource
- The community serves as an agent for the intervention (i.e. interventions working with already existing structures within the community)

We have focused on interventions in this category where the focus of the intervention is the community. The interventions described include community participatory research, like those described by Ragavan et al’s systematic review on community participatory research on domestic violence [44], as well as interventions that are ‘community placed’, where the community is a target of the intervention, and might not have been involved in the design of the intervention, in a participatory way.

All the interventions were focused on IPV reduction and improving social support and mental health outcomes for survivors of violence. Interventions like SASA [34, 39], used community members as a resource for the intervention. In the SASA intervention, community activists in the intervention sites were trained on GBV prevention, power inequalities and gender norms. After training, they carried out advocacy activities, engaging different stakeholders and members of their social networks to address harmful social norms around GBV. At the end of the intervention, there were reported lower rates of IPV among the intervention community. Other interventions like the ‘Framing Safety project’ [35], which focused on promoting agency and self-empowerment among survivors of violence, found that by providing means through which survivors of violence could tell their own stories and take ownership of this process, there was a resulting feeling of empowerment among the women. Other interventions used group therapy sessions that were community-based and culturally tailored to the specific target population. Wuest et al [41] described a collaborative partnership with different stakeholders (academic, NGOs and community members) to develop a comprehensive intervention to IPV, ‘Intervention for Health Enhancement After Leaving (iHEAL), a primary health care intervention for women recently separated from violent/abusive partners’. The post evaluation revealed significant reduction in depression and PTSD from baseline to 6 months post-intervention, these improvements in mental health outcomes, were present at 12 months post-intervention. Other outcomes, like social support, showed some initial improvement from baseline to 6 months post-intervention but these changes were not sustained till 12 months post-intervention.

### Community focused/ network interventions with a psychotherapy component

Three of the nine studies (1 RCT and 2 pre-post study) by Kelly et al [36], McWhirter et al [37], and Nicolaidis et al [38] described group therapy interventions that were designed in collaboration with the target population in a participatory way. These studies reported significant reductions in severity of mental health conditions like depression and PTSD, as well as an increase in social support and self-efficacy for the women who were involved in the study.

## Discussion

The focus of this systematic review was to assess the existing evidence available on IPV interventions focused on improving social support and/or mental health outcomes. To ensure that we included all relevant studies, we included both quantitative and qualitative articles. 27 articles were included in the systematic review out of 337 full text articles assessed. The following interventions were identified via the review: Survivor focused interventions (18 studies: 15 of these studies were focused on advocacy/case management services; 3 of these on advocacy/case management services with a psychotherapy component), community-based social support interventions (9 studies:4 out of these were community coordinated interventions with a psychotherapy component). The heterogeneity of the studies made it difficult to conduct a meta-analysis because of the variability in outcome measures, study design and processes and duration of interventions implemented. Survivor focused advocacy/case management IPV interventions made up most of the interventions identified (18 out of 27). The studies showed good to moderate evidence of the positive impact of these interventions on mental health outcomes and also access to social support for the IPV survivors included in the study, and in a few studies, a reduction in partner aggression or experience of IPV (IPV scores) [15–23]. In one study, by De Prince et al [42], where a community-based advocacy intervention was compared to an advocacy intervention that was focused on referral, both groups showed improvement in mental health outcomes, but the community-based advocacy intervention group (outreach) had slightly better mental health outcomes. A specific approach of the intervention was that it was community-led/ coordinated, the community based organisation reached out directly to the survivors of violence based on information from the systems based advocate, hence removing the need for survivors to seek out services themselves based on the referrals received from the system based advocate. This study might have important lessons for future advocacy interventions, as just provision of referrals might not ensure uptake of services, and a community coordinated follow up of IPV survivors might be more effective in ensuring uptake. However, it must be noted that only few of the advocate-based studies and 1 of the community-focused interventions reported an impact on IPV, with good level of evidence [15, 20–23, 34], similar to what has been found in other reviews of advocate-based interventions on intimate partner violence [45]. Tiwari et al’s study, which focused on the use of an empowerment, social support and advocacy-focused telephone intervention, found improved mental health outcomes among the intervention group. In comparison, Cripe et al’s [46] study also compared the effect of an empowerment-based intervention in comparison to usual care among abused pregnant women and found higher scores of improved safety behaviours among the intervention group compared to the control group but ‘no statistically significant difference in health-related quality of life, adoption of safety behaviours, and use of community resources between women in the intervention and control groups’. These differences we attribute to the study design, context and characteristics of the study participant. Goodman et al has described the importance of integrating a ‘social network’ approach into IPV interventions, and linking interventions with social networks of IPV survivors to ensure sustained access to social support for the survivors [9, 47]. Many of the advocacy/case management interventions described above have created these linkages by assisting IPV survivors identify sources of support within their existing networks and also engage in forming new social relationships [16, 18, 48]. However, more IPV interventions should integrate this approach in a coordinated systemic manner, as engaging with social network members of the IPV survivors ensures sustainability of the programme’s effects over time [9].

Several of the studies focused on psychotherapy interventions, which were individual, or group based. We classified these interventions separately as these interventions combined community-based advocacy with a therapeutic component, as opposed to advocacy/case management alone or community focused interventions. These interventions either used interpersonal therapy [31], traumatic treatment therapy [33], empowerment based group therapy [32], and a multicomponent intervention that combined therapeutic education sessions with information on resources and legal help remotely or ‘face to face’ [29]. All the interventions showed some impact on mental health outcomes and social support, with a weaker level of evidence of an impact on IPV. Although Zlotnick et al’s study[31] on a therapeutic intervention for pregnant IPV survivors, described an improvement of mental health outcomes (moderate effect on PTSD and depression), this finding was not sustained in the postpartum period, drawing attention to the need to assess the efficacy of interventions in this particular group, taking into account time dependent factors and participant attributes. A review done by Trabold et al [49], found that clinically focused interventions and group-based cognitive or cognitive behavioural interventions had a significant effect on depression and PTSD, as well as the uses of Interpersonal therapy (time dependent). However, as our review focused on therapies focused on improving social support and mental health outcomes, we included fewer studies. Although we found a similar trend as described by Trabold et al, among community-based interventions (including those that were psychotherapy focused), we could not assign the effect specifically to the type of psychotherapy method, but rather to the length, associated support services and context of the intervention. Sullivan et al [50] discussed the positive effect of trauma informed practice on mental health outcomes of IPV survivors in Shelters, showing evidence of the importance of IPV interventions to include a comprehensive ‘therapeutic or mental health component’. They also discussed the six components of what ‘trauma informed practice’ which includes: (a) reflecting and understanding of trauma and its many impacts on health and behaviour, (b) addressing both physical and psychological safety concerns, (c) using a culturally informed strengths-based approach, (d) helping to illuminate the nature and impact of trauma on survivors’ everyday experience, and (e) providing opportunities for clients to regain control over their lives’. These components were useful for advocacy/case management interventions for IPV survivors, to ensure a focus on improving mental health outcomes, intersectional collaboration between stakeholders, and that the intervention is survivor-centred and addresses cultural factors.

Interventions that compared remote and ‘face to face’ methods of support and advocacy mostly resulted in a reduction in IPV victimization and increased access to social support. In cases where different modes of intervention delivery were tested, for example a comparison between remotely delivered interventions (telephone or online) and ‘face to face’ interventions, no difference was noted between both modes of intervention. Krasnoff and Moscati’s study [51] discussed a multi-component referral, support and case management intervention that reported similar reduction in perceived IPV victimization as seen in studies included in our review. There were some differences in the telephone support interventions included, Stevens et al’s study [27] reported no difference in mental health outcomes compared to Tiwari et al’s study[15] which found an improvement in mental health outcomes among the intervention group. We postulate differences in outcome could be attributable to the fact that Tiwari’s intervention was more advocacy, empowerment and support focused than the intervention described in Stevens et al study, which was more information and referral focused.

### Summary of key findings and recommendations

- Most of the interventions that used advocacy with strong community linkages and a focus on community networks showed significant effects on mental health outcomes and access to social support, we assume a reason for this could be that because these interventions were rooted in the community, there were more sources of support that allowed the survivors of violence to develop better coping strategies, for example in the SASA study that included a strong community engagement component, community responses to cases of IPV were supportive of the survivor, and this had an effect on incidence of IPV. Future research and interventions on IPV should focus on ensuring stronger community linkages and outreach programmes to enhance the impact of the interventions on IPV survivors.
- This review found that when remote modes of intervention delivery were compared to ‘in person’ delivery of an intervention, there were no significant differences in outcome. This finding is of specific importance to hard-to-reach and vulnerable populations whom might be unwilling to access care at hospitals and registered clinics. More research focused on the use of remote support interventions among vulnerable populations (specifically IPV survivors), should be encouraged.
- There was a lot of heterogeneity in outcome measurements, especially measures of social support, drawing attention to the need for research and discussions around standardization and synthesis of evidence-based research on social support and IPV.
- In some of the studies, the ‘dosage of the intervention’, as well as some participant characteristics like age or ethnicity are often cited as potential moderators of some of the outcomes, more research on IPV intervention should examine the time dependent nature of interventions and their effect on outcomes similar to what was done by Bybee et al[16].

### Limitations

Although there were no language restrictions included in our search strategy, most of the studies retrieved and subsequently reviewed were in English, which could have influenced some of our conclusions.

## Conclusions

This systematic review presented the findings from IPV interventions focused on social support and mental health outcomes for IPV survivors. Advocacy/case management interventions that had strong linkages with communities, and were community focused seemed to have significant effects on mental health outcomes and access to resources for IPV survivors. However, all IPV survivors are not the same, and culture, socioeconomic background and the perception of abuse by the IPV survivor, have a mediating effect on their decision to access social support and utilize referral services. ‘An intersectional trauma informed practice’[50] [52] that addresses psychological and physical effects of IPV, is culturally appropriate and is empowering for the survivor, in addition to a ‘social network oriented approach’ might provide a way to ensure that IPV interventions are responsive to the needs of the IPV survivor[47]. This will ensure the interventions are targeted at ensuring survivors are able to access social support from their existing networks or new social relationships, and might also promote community education about IPV and promote community support for IPV prevention and mitigation. Future studies on IPV interventions should assess how these approaches impact the incidence of IPV, social and mental health outcomes across different populations’ of IPV survivors.

## Supporting information

S1 Checklist PRISMA 2009 checklist. (DOCX)
